# Extracellular Vesicles in the Pathogenesis, Clinical Characterization, and Management of Dermatomyositis: A Narrative Review

**DOI:** 10.3390/ijms25041967

**Published:** 2024-02-06

**Authors:** Cristina Ricco, Ahmed Eldaboush, Ming-Lin Liu, Victoria P. Werth

**Affiliations:** 1 Corporal Michael J. Crescenz Veterans Affairs Medical Center, Philadelphia, PA 19104, USA; car419@rwjms.rutgers.edu (C.R.); ahmed.eldaboush@pennmedicine.upenn.edu (A.E.); lium1@pennmedicine.upenn.edu (M.-L.L.); 2 Department of Dermatology, School of Medicine, University of Pennsylvania, Philadelphia, PA 19104, USA

**Keywords:** extracellular vesicles, microparticles, exosomes, microvesicles, dermatomyositis, inflammatory myopathy, autoimmune disease

## Abstract

Extracellular vesicles (EVs) are lipid-bilayer particles secreted from cells that primarily assist in cell-to-cell communication through the content of their cargo, such as proteins and RNA. EVs have been implicated in the pathogenesis of various autoimmune diseases, including dermatomyositis (DM), an inflammatory autoimmune disease characterized by distinct cutaneous manifestations, myopathy, and lung disease. We sought to review the role of EVs in DM and understand how they contribute to the pathogenesis and clinical characterization of the disease. We summarized the research progress on EVs in dermatomyositis based on recent publications. EV cargoes, such as double-stranded DNA, microRNA, and proteins, contribute to DM pathogenesis and mediate the proinflammatory response and cytokine release through signaling pathways such as the stimulator of interferon genes (STING) pathway. These nucleic acids and proteins have been proposed as disease-specific, stable biomarkers to monitor disease activity and responses to therapy. They also correlate with clinical parameters, inflammatory markers, and disease severity scores. Furthermore, some markers show an association with morbidities of DM, such as muscle weakness and interstitial lung disease. The continued study of EVs will help us to further elucidate our understanding of dermatomyositis.

## 1. Introduction

Extracellular vesicles (EVs) are lipid-bilayer particles secreted from cells that mediate cell-to-cell communication in both physiologic and pathologic states [1]. EVs exhibit other functions as well, such as assisting in the disposal of metabolic waste [2] and initiating mineralization in vertebrate tissues, such as cartilage and bone [3]. Extracellular vesicles can be categorized based on their physical characteristics (such as small vs. large EVs, or low, medium, and high densities), biochemical components (surface marker expression), or how they originated (e.g., keratinocyte-derived EVs). Terms such as microvesicles and exosomes have been used to describe different types of EVs; however, the all-encompassing term “extracellular vesicle” is preferred since the defining terms of these subcategories are contested [4]. EVs are characterized by their contents, such as nucleic acids, proteins, and lipids, and have the potential to influence various cellular processes through the delivery of their cargo. They have been investigated in the context of the pathogenesis of various autoimmune diseases [5,6,7] and have shown promise for use as biomarkers in diagnosis and management [8,9].

Dermatomyositis (DM) is an autoimmune inflammatory disease characterized by symmetric, proximal muscle weakness and pathognomonic cutaneous manifestations, such as Gottron’s papules and a heliotrope rash. Risk factors include exposure to multiple environmental factors, such as ultraviolet radiation, herbal supplements, drugs, infections, and cigarette smoking [10,11,12]. Muscle biopsies, which are usually diagnostic, reveal perivascular and perimysial inflammatory infiltrates, perifasicular atrophy, and microangiopathy. Skin biopsies reveal lymphocytic infiltrates, increased dermal mucin, and vacuolar modification of the basal layer, all histopathologic changes that are also observed in cutaneous lesions of Systemic Lupus Erythematosus (SLE) patients [13]. DM is associated with an increased risk of malignancies and can also be complicated by rheumatologic, respiratory, esophageal, or cardiac manifestations. Ischemic heart disease, interstitial lung disease, and malignancies are the most common causes of death in DM. Despite attempts to treat DM, the disease typically remains chronic, and therapies are focused on the management of symptoms.

The exact pathogenesis of DM is yet to be confirmed, but the two leading DM pathogenesis models concern type 1 interferon (IFN)-inducible gene products and the complement system. The role of EVs in these pathways has recently been highlighted in the literature [8,14,15]. Understanding how EVs may be contributing to the proinflammatory response in DM helps in determining the mechanisms behind the vasculopathy, muscle damage, and skin lesions that characterize this disease. Another important aspect of EVs in DM concerns their clinical applications, whereby EVs have shown potential for use as biomarkers in diagnosis, classification of subtypes, and management [7,8,14,15,16,17,18,19,20,21]. In addition, EVs have demonstrated significant associations with laboratory parameters such as C-reactive protein levels (CRP) and erythrocyte sedimentation rate (ESR) and clinical parameters such as disease severity, muscle strength, and skin inflammation in DM [8,14,19,20]. Here, we provide a narrative review in which we explore what is currently known about the role of EVs in the pathogenesis, clinical characterization, and management of DM.

## 2. Role of Extracellular Vesicles in the Pathogenesis of Dermatomyositis

### 2.1. The Type 1 Interferon Pathway

**Figure 1:**
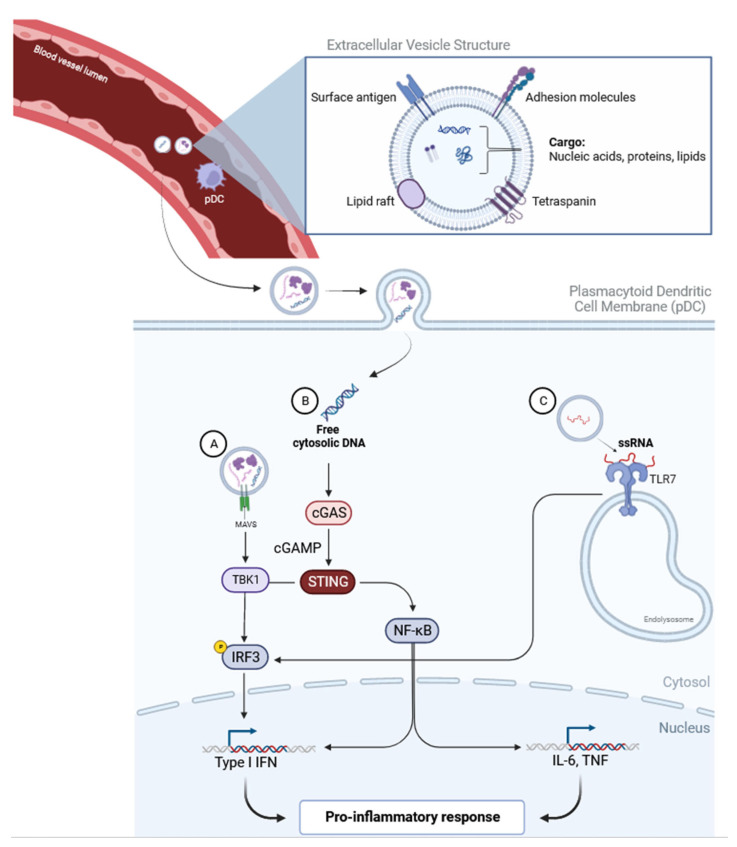
EVs in the type 1 IFN pathway. (**A**) Through an association with mitochondrial antiviral signaling protein (MAVS), which is depicted as an EV surface protein marker, studies have shown an upregulation of the type 1 IFN pathway. (**B**) Through direct endocytosis of EV cargo, the type 1 IFN pathway can be stimulated. (**C**) EVs released from infected cells can activate pDCs via interaction with Toll-like receptor 7 (TLR7) and produce type 1 IFNs [34,35].

There are a few theories that have been proposed to explain the pathogenesis of dermatomyositis. One mechanism involves the most upregulated pathway in DM, the type 1 interferon (IFN) pathway, specifically IFNβ [22,23]. Various immunogenic pathways are triggered that lead to downstream type 1 IFN production when the innate immune system is activated by foreign pathogens through pattern-recognition receptors, such as Toll-like receptors, RIG-I-like receptors, and cytoplasmic DNA sensors [24]. Increased IFN-inducible gene and protein expression has been identified in DM muscle, skin, and blood and contributes to muscle fiber, endothelial cell, and keratinocyte damage [25,26]. Significantly higher levels of IFN have been discovered in the plasma of DM patients compared to healthy controls (HC) and even compared to other inflammatory myopathies [27]. There is much evidence highlighting the role of EVs in the IFN pathway, such as the activation of Toll-like receptors by EV nucleic acid cargoes, thereby initiating the IFN response in the setting of infection (Figure 1) [28]. EVs from hepatitis C-permissive cells can trigger the innate immune system through the packaging and delivery of viral RNA to plasmacytoid dendritic cells (pDCs), resulting in the stimulation of the IFN response and aiding in host defense [29]. EVs have also been shown to directly aid in antiviral resistance through IFNα signaling, independent of direct cell-to-cell contact, in HBV-infected cells [30]. In our recent work, we found that mitochondrial antiviral signaling protein (MAVS), a protein that mediates antiviral innate immunity, has been shown to associate with plasma EVs, which can activate IFNβ production from pDCs, which is one possible way by which EVs are involved in DM pathogenesis through the induction of type 1 IFN [31]. Some studies have suggested dysregulated mitochondrial gene expression as an inciting factor in the upregulation of the IFN pathway [32]. Further study is needed on the hypothesis that the innate immune system inappropriately recognizes mitochondrial DNA as immunogenic, triggering a proinflammatory response that then drives the IFN type 1 pathway [33].

### 2.2. The Complement Pathway

Another proposed mechanism involves the pathological activation of the classical complement pathway, whereby C1q is activated, notably without immunoglobulin IgG deposits, by injured endothelial cells. This facilitates the formation of the membrane attack complex (MAC) [36,37]. The MAC deposits on endothelial cells of endomysial capillaries and destroys the microvasculature, causing ischemia and ultimately muscle atrophy [38]. The trigger for microvascular injury is still unclear; however, there are models suggesting that IFNs promote the classical complement pathway and vice versa, sustaining endothelial damage [8,37]. This mechanism is also related to the coagulation cascade pathway, whereby dysregulation of the complement cascade activates coagulation proteins [8,37]. As EVs have been implicated in the pathogenesis of multiple autoimmune diseases, a recent study sought to characterize the protein profile of plasma EVs in DM patients [8]. Biological process analysis on these DM plasma EVs revealed an enrichment of both coagulation and complement proteins, including fibrinogen alpha chain (FGA), fibrinogen beta chain (FGB), fibrinogen gamma chain FGG, von Willebrand factor (VWF), and complement proteins C1QB and C1QC compared to HCs [8]. Evidence also shows EVs have the potential to augment the complement cascade through binding of complement factors and proteins [39]. In addition, cells can also eliminate the MAC through membrane vesiculation and hence through the formation of EVs [40].

### 2.3. Vasculopathy, Muscle Damage, and the Proinflammatory Response

DM is characterized by inflammation of the vasculature resulting in defining cutaneous and muscular manifestations. Some of the latest research seeks to elucidate how EVs may contribute to the vasculopathy of DM [17]. One group hypothesized using circulating endothelial cells (CECs) and CEC-derived EVs to monitor vasculopathic involvement in juvenile DM (JDM) [17]. CECs detach from the vessel wall in response to endothelial injury [19,41,42,43], and significantly elevated concentrations have been observed in JDM patients compared to HCs, as well as in clinically active JDM compared to inactive JDM (disease activity was defined by the Pediatric Rheumatology International Trials Organization criteria) [17]. When looking at plasma CEC-derived EVs specifically, a strong association was found between total EV counts and endogenous thrombin potential (ETP), a parameter of thrombin generation and hence vascular injury [17]. The total counts of plasma EVs, endothelial-derived EVs, monocyte-derived EVs, Tissue Factor-positive EVs, and B-cell-derived EVs, which are all prothrombotic [44], were associated with ETP, but T-cell-derived EVs and platelet-derived EVs were not [17]. Previous studies from this group also revealed that there is increased plasma EV-mediated thrombin generation in children with vasculitis [45]. These results suggest that these EVs might be contributing to the occlusive vasculopathy and hypercoagulability seen in juvenile DM [17].

The stimulator of interferon genes (STING) pathway is also of interest in DM due to its critical role in type 1 IFN signaling and in the pathophysiology of many autoimmune diseases such as Lupus Erythematosus [46]. One recent study sought to investigate whether the STING pathway is involved in DM pathogenesis [14]. This study isolated “small” versus “large” EVs by centrifuging at two different speeds in order to study the functional differences in these two populations [14]. Human plasma-derived DM small EVs triggered significantly more proinflammatory cytokine release and induced more STING phosphorylation in peripheral blood mononuclear cells compared to HC EVs [14]. In addition, selective inhibition of this pathway through STING antagonists suppressed the proinflammatory effects seen with DM small EVs. These findings also corroborate a prior study [46] that linked serum-derived EVs to the production of type 1 IFNs through the STING pathway in SLE patients, supporting the overall notion that EVs are contributors to the pathogenesis of autoimmune diseases like DM [47]. Li et al. also showed that through the inhibition of TBK1, a downstream protein kinase in the STING pathway, the proinflammatory effects of DM plasma-derived EVs were inhibited [14]. Other studies have linked TBK-1 to the activation of the NF-Kβ, which enhances STING signaling [47]. Protein enrichment analysis performed by Meng et al. also supported these findings with enrichment of the NFKβ signaling pathway in plasma-derived EVs of DM patients [8]. Li et al. also observed an attenuated STING-mediated proinflammatory response through the digestion of DM EV cargo, specifically double-stranded DNA (dsDNA), emphasizing the importance of EVs as important catalysts and messengers in these complex pathways [14].

## 3. Characterization of Extracellular Vesicles in Dermatomyositis

The EV profile in DM patients differs from that of healthy controls in several ways, such as size and morphology; it has been shown that DM plasma-derived EVs have a smaller mean size [14] and amorphous structure compared to the typical round structure seen in HCs when imaged with transmission electron microscopy [19]. 

As for surface marker expression, EVs can express various proteins, such as the tetraspanin glycoproteins CD9, CD63, and CD9 [4]. Total human DM plasma-derived EVs have a more complex surface marker expression (triple and double marker expression) compared to total HC plasma-derived EVs, but there was no difference in the expression of these surface markers when comparing the same number of “small” EVs between the groups [14]. 

In terms of EV plasma concentration, the work by Shirafuji et al. was one of the earliest studies to compare EVs between DM patients and HCs [20]. Platelet-derived EVs (PDEVs) were significantly higher in DM patients compared to HCs [20]. Furthermore, PDEVs were significantly elevated in the active, untreated group compared to the inactive treatment group [20]. Other studies have corroborated these findings in DM and JDM patients [14,17]. In another study, CD3+, CD14+, and CD19+ EVs were found to be significantly higher in the plasma of DM patients compared to HCs, suggesting that they originated mainly from T lymphocytes, monocytes, and B lymphocytes, respectively [19]. It was established that these immune cells are implicated in the pathology of DM and were found in endomysial and perimysial infiltrates in the skeletal muscles of patients with DM [48,49]. Because creatine kinase (CK) is a muscle enzyme that is released from damaged muscles and is therefore typically elevated in the serum of DM patients [50], these authors also sought to investigate whether these EVs would be tagged with CK, but ultimately, the blood of DM patients did not reveal any CK+ EVs [19]. In terms of EV cargo and intra-vesicular content, distinct nucleic acid and protein profiles have been identified and are heavily explored later in this review.

## 4. EV-Associated Cargoes in DM

Here, we review EV-associated cargoes and their functions in DM.

### 4.1. EV-Associated Protein Cargoes

Using EVs as biomarkers arose from the idea that it is easier to detect circulating proteins in plasma compared to nucleic acids and EVs express abundant surface marker proteins [51]. Additionally, EV surface proteins are altered in pathological states and therefore would be an attractive choice for a disease marker [51]. 

As mentioned previously in the pathogenesis section, Meng et al. identified differentially expressed EV proteins (DEPs) in the plasma of DM patients, and analysis revealed an enrichment of both coagulation and complement proteins, linking these pathways to DM pathogenesis [8]. Similarly, Uto et al. identified the membrane proteins Plexin D1 (PLXD1), Cytochrome b-245 heavy chain (CYBB), solute carrier family 1 member 2 (SLCIA2), neogenin (NEO1), and intracellular adhesion molecule 5 (ICAM5) as being significantly upregulated in the serum EVs of DM patients compared to HCs and patients diagnosed with Rheumatoid Arthritis (RA), Systemic Lupus Erythematosus (SLE), Systemic Sclerosis (SSc), Duchenne muscular dystrophy (DMD), and Becker Muscular Dystrophy (BMD) [7]. Of these five proteins, Plexin D1 was chosen for further analysis, as it is expressed on immune cells [52,53] and more importantly, on the muscle fibers of JDM patients [54]. Additionally, liquid chromatography–tandem mass spectrometry confirmed that the detected peptide fragment of Plexin D1 originates from the cytoplasmic domain of Plexin D1, suggesting an EV-derived origin rather than a soluble protein origin [7]. Previous studies indicated that activated Plexin D1 is implicated in the defective angiogenesis and vascular tone control in SSc patients [55], as well as vascular remodeling and chronic muscle inflammation in JDM [7,54]. In addition, as mentioned previously, EV-associated MAVS protein has shown a potential role in the pathogenesis of DM through the induction of type 1 IFN [31].

### 4.2. EV-Associated Nucleic Acid Cargoes

Exosomal RNAs are very stable [56,57,58], and so their potential to act as disease biomarkers is intriguing. Non-coding RNAs have been recently implicated in the pathogenesis of DM [59,60], particularly microRNA (miRNA) and long non-coding RNA (lncRNA), which we will discuss in the following section [10]. It is important to acknowledge that when isolating EVs from the plasma or serum, it is extremely challenging to isolate a 100% pure EV prep due to the technical limitations of the instruments and methods used to isolate EVs today [61]. The complexity involved in isolating EVs is attributed to the large quantity of nonvesicular extracellular nanoparticles (NVEPs) in the plasma and serum, such as exomeres, supermeres, argonaute protein complexes, vaults, albumin, and lipoproteins, which have been shown to carry nucleic acids [62,63,64,65,66,67,68]. NVEPs are isolated in a similar fashion to EVs, that is, by ultracentrifugation at very high speeds, and are within similar size ranges to EVs, which makes them a potential contaminant in EV preparations [69,70,71]. Because of this potential for contamination, one may question whether nucleic acids isolated from EV preparations are truly derived from EVs alone. The articles selected for this review use EV isolation methods that are up to standard according to the Minimal information for studies of extracellular vesicles (MISEV) guidelines. In this review, we aim to summarize all the current knowledge as it relates to EVs and dermatomyositis as well as inform the reader on this issue between EVs and NVEPs. As the study of EVs is still an intense field of research, further investigation is needed to distinguish the roles of EVs and NVEPs as carriers of genetic material.

#### 4.2.1. EV-Associated MicroRNA (miRNA)

Jiang et al. uncovered ten differentially expressed miRNAs in patients with JDM compared to healthy controls, eight of which were upregulated and two were downregulated [18]. They also incubated JDM plasma EVs with human aortic endothelial cells and observed an altered transcriptional gene profile. A functional enrichment analysis revealed 59 differentially expressed genes (DEGs) with altered expression levels compared to control HAECs. The biological functions that correlate with the lower-expressed genes include cell migration, intercellular junction assembly, cell–cell adhesion, and cytoplasm organization and biogenesis, which are basic endothelial cell functions [18]. Overall, this analysis revealed an altered transcriptional profile that could be attributed to JDM EVs, suggesting that these genes contribute to the vasculopathy and endothelial injury in JDM [17].

Zhong et al. compared the plasma EV cargo from 10 DM patients with the Anti-Melanoma Differentiation-Associated Protein 5 Antibody-Positive subtype with complications of interstitial lung disease (ILD-MDA5 Ab+) and also compared their plasma EVs to those of healthy controls [16]. Analysis revealed upregulation of 38 microRNAs (miRNAs) and downregulation of 21 microRNAs. Furthermore, DM patients who tested negative for 16 different myositis-specific antigens (MSAs) and had no interstitial lung disease (nonILD-MSA16-) had 73 differentially expressed miRNAs compared to healthy controls. Two miRNAs, Homo sapiens *(hsa)-miR-4488* and *hsa-miR-1228-5p*, were significantly upregulated in the ILD-MDA5 Ab+ subset, and bioinformatic analysis suggested that these miRNAs may contribute to DM pathogenesis through their target genes, which are involved in systemic inflammation through the NFKβ pathway [16].

Two other studies additionally identified RNA profiles from DM EVs, one study focusing on miRNAs from neutrophil-derived EVs and the other on those from plasma-derived EVs. The former study identified that a total of 32 miRNAs were differentially expressed in DM patients compared to healthy controls, 17 of which were upregulated and 15 were downregulated [21]. Gene ontology analysis suggested that these miRNA target genes participate in actin filament organization, endothelial cell development and differentiation, and muscle tissue development [21]. The latter study detected fifty-three differentially expressed miRNAs in DM patients compared to healthy controls, forty-four of which were upregulated and nine were downregulated [15]. Gene ontology analysis for this study suggested that these miRNA target genes participate in the autophagy pathway, the regulation of apoptotic signaling, the development of muscle tissue, and the Wnt signaling pathway [15]. Overall, these advanced analyses suggest key pathways and biological processes that should be further investigated in the context of EVs and their role in the pathogenesis of DM.

#### 4.2.2. EV-Associated Long Non-Coding RNA (lncRNA)

A study by Li et al. detected a total of 452 differentially expressed long non-coding RNAs (lncRNAs) in the plasma EVs of DM patients compared to healthy controls; 313 of these lncRNAs were upregulated while 139 were downregulated [15].

Another study revealed 379 differentially expressed lncRNAs from neutrophil-derived EVs in DM patients compared to healthy controls, 124 of which were upregulated, whereas 255 were downregulated [21]. Additionally, functional analysis of these DE lncRNAs suggested that they might participate in skeletal muscle cell proliferation and regulation of the production of IFNβ, processes central to DM pathogenesis [21].

#### 4.2.3. EV-Associated Messenger RNA (mRNA)

Li et al., 2022, also investigated mRNAs as a part of their RNA profiling and detected a total of 689 differentially expressed (DE) mRNAs from DM plasma-derived EVs compared to control EVs [15]. A total of 484 of them were downregulated and 205 were upregulated [15]. Bioinformatic analysis for these DE mRNAs revealed an enrichment in multiple pathways, some of which included the autophagy pathway and the response to IFN-γ [15].

#### 4.2.4. EV-Associated dsDNA

As mentioned previously, DM exosomal dsDNA plays an important role in the STING and type 1 IFN pathways [14]. Interestingly, both genomic and mitochondrial DNA can be carried in EVs, and dsDNA captured by EVs is seemingly expressed both intra-vesicularly and on the surface [72].

## 5. The Clinical Implications of Extracellular Vesicles in Dermatomyositis

The notion of EVs as disease-specific biomarkers has gained much attention in recent years. Here, we review the clinical importance of EVs and their potential for therapeutic use.

### 5.1. Extracellular Vesicles in Diagnosis and Prognosis of Dermatomyositis

#### 5.1.1. Association between Extracellular Vesicles and Laboratory Parameters or Inflammatory Markers in Dermatomyositis

Several studies have demonstrated a correlation between different types of EV cargo and various laboratory and clinical parameters (Table 1). For instance, C1QB and C1QC, two complement pathway-associated proteins, positively correlated with the serum levels of CRP, ESR, and platelet count in DM patients [8]. Von Willebrand factor (VWF), a protein that plays a pivotal role in platelet plug formation and vascular thrombosis [73], also positively correlated with serum ferritin and antinuclear antibody titers (ANA) [8]. VWF is a multimeric glycoprotein that is cleaved by ADAMTS13, facilitating its adhesion to platelets [55,74]. Interestingly, ADAMTS13 also positively correlated with ANA in DM patients [8]. Among the DEPs identified for these DM EVs, the acute-phase reactants serum amyloid A-1 protein (SAA1), haptoglobin, and SERPINA3 (α-1-antichymotrypsin) were positively correlated with clinical parameters as well [8]. More specifically, SAA1 was correlated positively with CRP and ESR, whereas SERPINA3 correlated positively with ESR [8]. However, none of the aforementioned proteins correlated with CK [8].

Another report showed that the number of platelet-derived EVs (PDEVs) correlated with levels of CRP in patients with DM. The PDEV/platelet ratio also correlated significantly with levels of CRP but not with levels of CK [20]. Similarly, Baka et al. reported no correlation between DM immune cell-derived EVs and CK [19]. Since DM is an inflammatory myopathy, it was essential to investigate how these enzymes correlate with plasma-derived EVs in DM.

Plexin D1, as mentioned previously, is another differentially expressed protein in the plasma EVs of DM patients [7]. The serum levels of CD9+ Plexin D1+ EVs were found to significantly correlate with serum levels of the muscle enzyme aldolase, white blood cell counts, neutrophil counts, and platelet counts as measures of disease activity and systemic inflammation in DM patients [7]. Again, there was no correlation with CK [7]. Interestingly, CD9+ Plexin D1+ EVs did not correlate with the muscle enzyme aldolase in patients with DMD [7], a disease in which the hallmark is substantial muscle damage and significantly elevated levels of muscle enzymes [75]. This suggests that the correlation between Plexin D1 and aldolase is specific to DM patients [7]. Moreover, CD9+ Plexin D1+ EVs did not correlate with the level of inflammatory markers (WBCs, platelets, and neutrophils) of other autoimmune diseases, namely, RA, SLE, and SSC [7]. Taken together, Plexin D1 has been proposed as a specific biomarker for DM disease activity [7].

In a study by Li et al., 2022, a few differentially expressed miRNAs in plasma-derived EVs of DM patients were proposed as potential disease biomarkers [15]. The plasma levels of *hsa-miR-125a-3p* were positively correlated with the concentrations of serum aspartate aminotransferase (AST), aminotransferase (ALT), and lactate dehydrogenase (LDH) [15]. These miRNA levels were also negatively correlated with the disease duration and the absolute neutrophilic count [15]. The miRNA *hsa-miR-1246* negatively correlated with the number of monocytes and *hsa-miR-3614-5p* expression positively correlated with AST and ILD [15]. Notably, *hsa-miR-125a-3p* had the strongest correlation (with AST, ALT, and LDH) among other miRNAs [15]. It is also important to note that none of the aforementioned miRNAs significantly correlated with CK [15]. Although EV-miRNAs correlated with various clinical and laboratory parameters, five different studies revealed no correlation with the muscle enzyme CK, even in the presence of muscle weakness [7,8,15,19,20]. Larger studies are needed to validate these markers and further investigate their clinical utility.

#### 5.1.2. Association with Different Dermatomyositis Subsets

There has been increasing recognition surrounding the classification of DM, as this disease can be further categorized into various subsets based on clinical and laboratory characteristics [76]. Myositis-specific autoantibodies (MSAs) play a pivotal role in defining subsets of DM through serology [77]. Testing for MSAs can be useful in predicting the prognosis and pattern of organ involvement in DM [76]. As discussed, various EV components have been proposed as disease-specific biomarkers [7,8,15,16,21] and so it follows that they may have the potential to distinguish DM subgroups.

The presence of anti-Jo-1 (anti-histidyl-tRNA synthetase) antibodies typically represents a more severe subset of DM, known as anti-synthetase syndrome, and is usually associated with a worse prognosis and severe symptoms such as interstitial lung disease [78,79]. One study discovered a significantly higher number of T-lymphocyte-, B-lymphocyte-, and monocyte-derived EVs in anti-Jo-1-positive DM patients compared to anti-jo-1-negative DM patients [19]. Additionally, DM patients with lung disease were revealed to have a significantly higher number of lymphocyte- and monocyte-derived EVs compared to DM patients without lung disease [19].

Anti-MDA5-positive serology determines a subset of DM that is usually associated with a more severe course of interstitial lung disease that can be rapidly progressive, requiring intensive immunosuppressive therapy [80,81,82]. A comparative analysis of plasma EVs from DM patients revealed that 51 exosomal miRNAs were significantly upregulated in DM patients with ILD who were also positive for MDA5 (DM-ILD-MDA5+), compared to DM patients without ILD who were negative for 16 different myositis-specific antibodies (DM-nonILD-MSA16-) [16]. Thirty-three miRNAs were also discovered to be significantly downregulated in DM-ILD-MDA5+ patients compared to DM-nonILD-MSA16- patients [16]. The miRNAs *hsa-miR-1228-5p* and *hsa-miR-4488* in particular were significantly upregulated in DM-ILD-MDA5+ patients, and hsa-*miR-1228-5p* was significantly downregulated in DM-nonILD-MSA16- patients when compared to healthy controls [16].

In a different study, the level of expression of *hsa-miR-3614-5p* positively correlated with ILD and MDA5+ rates in DM patients. Moreover, *hsa-miR-1256* was positively correlated with the presence of anti-Mi2α antibodies [15].

Through proteomic analysis, Meng et al. found SERPINA3, a DEP in DM plasma-derived EVs, to be highly expressed in DM patients but also specifically in MDA5+ DM patients compared to MDA5- DM patients [8]. SERPINA3 is a serine protease inhibitor from the serpin superfamily that has been linked to a variety of biological activities such as inflammation [83] and activation of the complement system [83,84]. Urinary SERPINA3 was also proposed as a biomarker that correlates with the activity of lupus nephritis in SLE [85]. Mannose-binding lectin-associated serine protease 2 (MASP2) was also higher in DM MDA5+ patients compared to DM MDA5- patients [8]. MASP2 is a serine protease that is thought to be involved in complement activation and was also suggested as a marker for disease activity in SLE [86,87]. Additionally, the DEPs SAA1, Complement 9 (C9), Carboxypeptidase N2 (CPN2), and laminin subunit gamma 1 LAMC1) were also highly expressed in DM ILD+ patients compared to DM ILD- patients [8]. Furthermore, they identified the proteins SAA1 and S100A8 to be significantly more expressed in MDA5+ patients whose disease was complicated with ILD compared to MDA5+ patients who did not have ILD [8]. Of note, SAA1 and S100A8 were associated with interstitial lung disease in patients with DM in a previous study [5]. These results suggest unique patterns of protein and nucleic acid expression in the EVs of patients with different DM subtypes.

#### 5.1.3. Association with Disease Activity in Dermatomyositis

EVs have been proposed as biomarkers that correlate with disease activity and severity for various autoimmune diseases [3,4]. For instance, urinary exosomal miRNA *miR-146a* and serum exosomal *miR-451a* levels significantly correlated with disease activity and renal damage in patients with lupus nephritis [5,6]. Herein, we review possible EV markers for disease activity in DM.

#### 5.1.4. Involvement of Extracellular Vesicles in Myositis due to Dermatomyositis

Serum levels of Plexin D1 were significantly more elevated in DM patients suffering from muscle weakness or muscle pain compared to DM patients with no muscle symptoms [7]. Serum levels of Plexin D1 also distinguished DM patients from healthy controls and patients with RA and SLE with good diagnostic accuracy [7]. In addition, no difference in serum Plexin D1 levels was detected in DMD patients compared to healthy controls, and in fact, they were also significantly less than those of DM patients [7].

One study seemed to contradict the general findings that describe a positive correlation between the number of plasma EVs with the degree of muscle weakness [19]. Baka et al. reported that the number of monocyte-derived and B-lymphocyte-derived EVs correlated positively with muscle strength. This study used the manual muscle test (MMT) to clinically evaluate muscle strength.

#### 5.1.5. Extracellular Vesicles and Skin Inflammation in Dermatomyositis

Li et al. discovered that small EVs derived from the plasma of DM patients significantly correlated with the Cutaneous Dermatomyositis Disease Area and Severity Index (CDASI) score [14], a well-validated disease outcome measure to assess the activity and severity of cutaneous DM [88]. However, there was no association found between large EVs and the CDASI score [14].

#### 5.1.6. Diagnostic Predictability of Extracellular Vesicles in Diagnosis and Prognosis of Dermatomyositis

As discussed earlier, Plexin D1 exhibited good diagnostic accuracy in differentiating DM patients from healthy controls (AUC of 0.75), as well as from patients with other autoimmune diseases (AUC of 0.78) and patients with DMD/BMD (AUC of 0.74) in the receiver operating characteristic analyses (ROC) [7]. Another ROC analysis by Meng et al. revealed that 15 differentially expressed proteins in DM patients, mostly associated with the complement and coagulation cascade pathways (e.g., C1QC, SERPINA3, FGA, FGB, VWF), had the ability to distinguish DM patients from healthy controls with a high AUC [8]. The collective AUC increased to 0.97 with a sensitivity of 94% and a specificity of 100% [8].

Taken together, these differentially expressed proteins show promising diagnostic potential. However, bigger studies are needed to evaluate the feasibility of their use in the diagnosis of DM. 

### 5.2. Therapeutic Potential of Extracellular Vesicles in Dermatomyositis

The studies we have reviewed show that EVs exhibit a distinct profile according to the body’s disease state [7,8,14,15,16,17,18,19,20,21]. Further investigation into the role of EVs in DM will allow us to improve our surveillance of disease detection and progression. It may also help us monitor the response to different types of treatments and identify patterns of treatment resistance. For example, the initially elevated levels of platelet-derived EVs dropped significantly following treatment with glucocorticoids in previously untreated DM patients [20]. The number of CD9+ Plexin D1+ EVs also was significantly reduced after treatment in DM patients [7]. Interestingly, the levels of these EVs actually increased post-treatment in two-thirds of patients who did not respond to therapy [7].

In terms of the DM EV transcriptional profile, DEP SERPINA1 was downregulated in three out of four MDA5+DM patients treated with immunosuppressive medications compared to these patients pre-treatment [8]. This poses an interesting question where we can consider the potential of SERPINA1 to serve as a marker of treatment responsiveness in DM patients. Likewise, the DM EV DEP *hsa-miR-125a-3p* is another potential biomarker that was significantly decreased in patients post-treatment with immunosuppressive medications compared to these patients pre-treatment [15]. These correlations should be studied further to evaluate them for potential use in DM management and treatment.

## 6. Conclusions

In this review, we have discussed multiple aspects of EVs in the context of DM and their potential use in the diagnosis and therapeutics of this disease. Their ability to deliver cargo and hence mediate multiple pathways that are implicated in DM pathogenesis is noteworthy and can help us further elucidate the mysteries of this disease. Furthermore, their significant correlation with multiple laboratory and clinical parameters demonstrates a strong case for their use as disease-specific biomarkers.

One interesting point to consider when evaluating the published studies in this review is the methodologies for the isolation of EVs and the terminologies used to describe EVs. Per the most recently updated EV guidelines (Minimal information for studies of extracellular vesicles, MISEV2018) [4] published by the International Society for Extracellular Vesicles, to claim that you are studying a specific subpopulation of EVs such as exosomes and to attribute specific functions and characteristics to a subpopulation requires great precision and rigorous data. There is also no “gold standard” protocol to isolate EVs, as there are various methods of isolation, some more stringent than others, resulting in purer preparations and yet greater EV losses. Our review sought to investigate what is currently known about EVs in the pathogenesis and management of DM, and as this is a relatively newer field, there were few primary papers that we were able to evaluate. Therefore, we collectively referred to all the vesicles in these papers as EVs. Of note, some authors have isolated EVs from plasma and others from serum. Prior studies from our group amongst others have shown that EVs in the serum are not representative of the actual population of EVs in circulation due to clotting interactions involving EVs, the formation of apoptotic blebs released from activated platelets during clotting, and possible EV-associated protein cleavage due to the coagulation cascade [89,90].

Additionally, most of these studies were conducted on very small numbers of patients, so further investigation with larger cohorts will allow for the validation of these findings. Some of these studies also used immune cell-derived EVs as biomarkers, which are not specific to DM. Nonetheless, these preliminary studies have provided us with much exciting data to ponder and promising clinical correlations that should be further explored.

## Figures and Tables

**Table 1:** Correlation between EVs and laboratory parameters in dermatomyositis.

Laboratory Parameter	EV Component	Strength of Correlation (r)	References
CRP	C1QB	+0.648	Meng et al., 2022 [8]
	C1QC	+0.682	
	SAA1	+0.546	
ESR	C1QB	+0.611	
	C1QC	+0.628	
	SERPINA3	+0.657	
	SAA1	+0.523	
Platelet count	C1QB	+0.035	
	C1QC	+0.523	
	Plexin D1	+0.408	Uto et al., 2022 [7]
Ferritin	VWF	+0.673	Meng et al., 2022 [8]
ANA	ANGPTL6	+0.693	
	VWF	+0.740	
	COLEC11	+0.668	
Aldolase	Plexin D1	+0.481	Uto et al., 2022 [7]
WBCs count	Plexin D1	+0.381	
Neutrophils count	Plexin D1	+0.450	
AST	*hsa-miR-125a-3p*	+	Li et al., 2022 [15]
	*hsa-miR-3614*	+	
ALT	*hsa-miR-125a-3p*	+	
LDH	*hsa-miR-125a-3p*	+	

CRP: C-reactive protein; ESR: Erythrocyte sedimentation rate; ANA: Antinuclear antibodies; WBCs: White blood cells; AST: Aspartate aminotransferase; ALT: Alanine aminotransferase; LDH: lactate dehydrogenase; C1QB and C1QC: complement proteins C1QB and C1QC; SAA1: Serum amyloid A-1 protein; SERPINA3: α-1-antichymotrypsin; VWF: Von Willebrand factor; ANGPTL6: Angiopoietin-like 6; COLEC11: Collectin subfamily member 11; *hsa-miR*: Homo sapiens microRNA.

## References

[1] Liu M., Williams K.J., Werth V.P. (2016). Microvesicles in autoimmune diseases. Adv. Clin. Chem..

[2] Vidal M. (2019). Exosomes: Revisiting their role as “garbage bags”. Traffic.

[3] Plaut J.S., Strzelecka-Kiliszek A., Bozycki L., Pikula S., Buchet R., Mebarek S., Chadli M., Bolean M., Simao A.M.S., Ciancaglini P. (2019). Quantitative atomic force microscopy provides new insight into matrix vesicle mineralization. Arch. Biochem. Biophys..

[4] Théry C., Witwer K.W., Aikawa E., Andriantsitohaina R., Baharvand H., Bauer N.N., Baxter A.A., Beckham C., Bielska E., Boireau W. (2018). Minimal information for studies of extracellular vesicles 2018 (MISEV2018): A position statement of the International Society for Extracellular Vesicles and update of the MISEV2014 guidelines. J. Extracell. Vesicles.

[5] Perez-Hernandez J., Martinez-Arroyo O., Ortega A., Galera M., Solis-Salguero M.A., Chaves F.J., Redon J., Forner M.J., Cortes R. (2021). Urinary exosomal miR-146a as a marker of albuminuria, activity changes and disease flares in lupus nephritis. J. Nephrol..

[6] Tan L., Zhao M., Wu H., Zhang Y., Tong X., Gao L., Zhou L., Lu Q., Zeng J. (2021). Downregulated Serum Exosomal miR-451a Expression Correlates With Renal Damage and Its Intercellular Communication Role in Systemic Lupus Erythematosus. Front. Immunol..

[7] Uto K., Ueda K., Okano T., Akashi K., Takahashi S., Nakamachi Y., Imanishi T., Awano H., Morinobu A., Kawano S. (2022). Identification of plexin D1 on circulating extracellular vesicles as a potential biomarker of polymyositis and dermatomyositis. Rheumatology.

[8] Meng S., Wang T., Zhao Q., Hu Q., Chen Y., Li H., Liu C., Liu D., Hong X. (2022). Proteomics Analysis of Plasma-Derived Exosomes Unveils the Aberrant Complement and Coagulation Cascades in Dermatomyositis/Polymyositis. J. Proteome Res..

[9] Wu W., Song S., Zhang Y., Li X. (2020). Role of Extracellular Vesicles in Autoimmune Pathogenesis. Front. Immunol..

[10] Parks C.G., Wilkerson J., Rose K.M., Faiq A., Noroozi Farhadi P., Long C.S., Bayat N., Brunner H.I., Goldberg B., McGrath J.A. (2020). Association of Ultraviolet Radiation Exposure with Dermatomyositis in a National Myositis Patient Registry. Arthritis Care Res..

[11] Schiffenbauer A., Faghihi-Kashani S., O’hanlon T.P., Flegel W.A., Adams S.D., Targoff I.N., Oddis C.V., Ytterberg S.R., Aggarwal R., Christopher-Stine L. (2018). The Impact of Cigarette Smoking on the Clinical and Serological Phenotypes of Polymyositis and Dermatomyositis. Semin. Arthritis Rheum..

[12] Bax C.E., Maddukuri S., Ravishankar A., Pappas-Taffer L., Werth V.P. (2021). Environmental Triggers of Dermatomyositis: A Narrative Review. Ann. Transl. Med..

[13] Smith E.S., Hallman J.R., Deluca A.M., Goldenberg G., Jorizzo J.L., Sangueza O.P. (2009). Dermatomyositis: A Clinicopathological Study of 40 Patients. Am. J. Dermatopathol..

[14] Li Y., Bax C., Patel J., Vazquez T., Ravishankar A., Bashir M.M., Grinnell M., Diaz D., Werth V.P. (2021). Plasma-derived DNA containing-extracellular vesicles induce STING-mediated proinflammatory responses in dermatomyositis. Theranostics.

[15] Li L., Zuo X., Liu D., Luo H., Zhang H., Peng Q., Wang G., Zhu H. (2022). Plasma exosomal RNAs have potential as both clinical biomarkers and therapeutic targets of dermatomyositis. Rheumatology.

[16] Zhong D., Wu C., Xu D., Bai J., Wang Q., Zeng X. (2021). Plasma-Derived Exosomal hsa-miR-4488 and hsa-miR-1228-5p: Novel Biomarkers for Dermatomyositis-Associated Interstitial Lung Disease with Anti-Melanoma Differentiation-Associated Protein 5 Antibody-Positive Subset. BioMed Res. Int..

[17] Papadopoulou C., Hong Y., Krol P., Al Obaidi M., Pilkington C., Wedderburn L., Brogan P.A., Eleftheriou D. (2021). The Vasculopathy of Juvenile Dermatomyositis: Endothelial Injury, Hypercoagulability, and Increased Arterial Stiffness. Arthritis Rheumatol..

[18] Jiang K., Karasawa R., Hu Z., Chen Y., Holmes L., O’Neil K.M., Jarvis J.N. (2019). Plasma exosomes from children with juvenile dermatomyositis are taken up by human aortic endothelial cells and are associated with altered gene expression in those cells. Pediatr. Rheumatol..

[19] Baka Z., Senolt L., Vencovsky J., Mann H., Simon P.S., Kittel Á., Buzás E., Nagy G. (2010). Increased serum concentration of immune cell derived microparticles in polymyositis/dermatomyositis. Immunol. Lett..

[20] Shirafuji T., Hamaguchi H., Higuchi M., Kanda F. (2009). Measurement of platelet-derived microparticle levels using an enzyme-linked immunosorbent assay in polymyositis and dermatomyositis patients. Muscle Nerve.

[21] Li L., Zuo X., Liu D., Luo H., Zhu H. (2021). The Functional Roles of RNAs Cargoes Released by Neutrophil-Derived Exosomes in Dermatomyositis. Front. Pharmacol..

[22] Huard C., Gullà S.V., Bennett D.V., Coyle A.J., Vleugels R.A., Greenberg S.A. (2017). Correlation of cutaneous disease activity with type 1 interferon gene signature and interferon β in dermatomyositis. Br. J. Dermatol..

[23] Wong D., Kea B., Pesich R., Higgs B.W., Zhu W., Brown P., Yao Y., Fiorentino D. (2012). Interferon and Biologic Signatures in Dermatomyositis Skin: Specificity and Heterogeneity across Diseases. PLoS ONE.

[24] Gürtler C., Bowie A.G. (2013). Innate immune detection of microbial nucleic acids. Trends Microbiol..

[25] Greenberg S.A., Pinkus J.L., Pinkus G.S., Burleson T., Sanoudou D., Tawil R., Barohn R.J., Saperstein D.S., Briemberg H.R., Ericsson M. (2005). Interferon-α/β–mediated innate immune mechanisms in dermatomyositis. Ann. Neurol..

[26] Greenberg S.A. (2010). Dermatomyositis and Type 1 Interferons. Curr. Rheumatol. Rep..

[27] Salajegheh M., Kong S.W., Pinkus J.L., Walsh R.J., Liao A., Nazareno R., Amato A.A., Krastins B., Morehouse C., Higgs B.W. (2010). Interferon-stimulated gene 15 (ISG15) conjugates proteins in dermatomyositis muscle with perifascicular atrophy. Ann. Neurol..

[28] Assil S., Webster B., Dreux M. (2015). Regulation of the Host Antiviral State by Intercellular Communications. Viruses.

[29] Dreux M., Garaigorta U., Boyd B., Décembre E., Chung J., Whitten-Bauer C., Wieland S., Chisari F. (2012). Short-Range Exosomal Transfer of Viral RNA from Infected Cells to Plasmacytoid Dendritic Cells Triggers Innate Immunity. Cell Host Microbe.

[30] Li J., Liu K., Liu Y., Xu Y., Zhang F., Yang H., Liu J., Pan T., Chen J., Wu M. (2013). Exosomes mediate the cell-to-cell transmission of IFN-α-induced antiviral activity. Nat. Immunol..

[31] Li Y., Zeidi M., Bashir M.M., Werth V.P., Liu M. (2019). Extracellular MAVS associates with microvesicles that can actively trigger IFNβ production. J. Investig. Dermatol..

[32] West A.P., Khoury-Hanold W., Staron M., Tal M.C., Pineda C.M., Lang S.M., Bestwick M., Duguay B.A., Raimundo N., MacDuff D.A. (2015). Mitochondrial DNA stress primes the antiviral innate immune response. Nature.

[33] Wilkinson M.G.L., Moulding D., McDonnell T.C.R., Orford M., Wincup C., Ting J.Y.J., Otto G.W., Restuadi R., Kelberman D., Papadopoulou C. (2023). Role of CD14+ monocyte-derived oxidised mitochondrial DNA in the inflammatory interferon type 1 signature in juvenile dermatomyositis. Ann. Rheum. Dis..

[34] (2023). Adapted from “cGAS-STING DNA Detection”, by BioRender.com. https://app.biorender.com/biorender-templates.

[35] (2023). Adapted from “Extracellular Vesicles”, by BioRender.com. https://app.biorender.com/biorender-templates.

[36] Dalakas M.C., Alexopoulos H., Spaeth P.J. (2020). Complement in neurological disorders and emerging complement-targeted therapeutics. Nat. Rev. Neurol..

[37] Lahoria R., Selcen D., Engel A.G. (2016). Microvascular alterations and the role of complement in dermatomyositis. Brain.

[38] Dalakas M.C. (2022). Complement in autoimmune inflammatory myopathies, the role of myositis-associated antibodies, COVID-19 associations, and muscle amyloid deposits. Expert Rev. Clin. Immunol..

[39] Buzas E.I., Toth E.A., Sodar B.W., Szabo-Taylor K.E. (2018). Molecular interactions at the surface of extracellular vesicles. Semin. Immunopathol..

[40] Pilzer D., Gasser O., Moskovich O., Schifferli J.A., Fishelson Z. (2005). Emission of membrane vesicles: Roles in complement resistance, immunity and cancer. Semin. Immunopathol..

[41] Figarella-Branger D., Schleinitz N., Boutière-Albanèse B., Camoin L., Bardin N., Guis S., Pouget J., Cognet C., Pellissier J., Dignat-George F. (2006). Platelet-endothelial cell adhesion molecule-1 and CD146: Soluble levels and in situ expression of cellular adhesion molecules implicated in the cohesion of endothelial cells in idiopathic inflammatory myopathies. J. Rheumatol..

[42] Walsh R.J., Kong S.W., Yao Y., Jallal B., Kiener P.A., Pinkus J.L., Beggs A.H., Amato A.A., Greenberg S.A. (2007). Type I interferon–inducible gene expression in blood is present and reflects disease activity in dermatomyositis and polymyositis. Arthritis Rheum..

[43] Rigolet M., Hou C., Baba Amer Y., Aouizerate J., Periou B., Gherardi R.K., Lafuste P., Authier F.J. (2019). Distinct interferon signatures stratify inflammatory and dysimmune myopathies. RMD Open.

[44] Falati S., Liu Q., Gross P., Merrill-Skoloff G., Chou J., Vandendries E., Celi A., Croce K., Furie B.C., Furie B. (2003). Accumulation of Tissue Factor into Developing Thrombi In Vivo Is Dependent upon Microparticle P-Selectin Glycoprotein Ligand 1 and Platelet P-Selectin. J. Exp. Med..

[45] Eleftheriou D., Hong Y., Klein N.J., Brogan P.A. (2011). Thromboembolic disease in systemic vasculitis is associated with enhanced microparticle-mediated thrombin generation. J. Thromb. Haemost..

[46] Kato Y., Park J., Takamatsu H., Konaka H., Aoki W., Aburaya S., Ueda M., Nishide M., Koyama S., Hayama Y. (2018). Apoptosis-derived membrane vesicles drive the cGAS–STING pathway and enhance type I IFN production in systemic lupus erythematosus. Ann. Rheum. Dis..

[47] Abe T., Barber G.N. (2014). Cytosolic-DNA-Mediated, STING-Dependent Proinflammatory Gene Induction Necessitates Canonical NF-κB Activation through TBK1. J. Virol..

[48] Pedrol E., Grau J.M., Casademont J., Cid M.C., Nasanes F., Fernandez-Sola J., Urbano-Marquez A. (1995). Idiopathic inflammatory myopathies. Immunohistochemical analysis of the major histocompatibility complex antigen expression, inflammatory infiltrate phenotype and activation cell markers. Clin. Neuropathol..

[49] Arahata K., Engel A.G. (1984). Monoclonal antibody analysis of mononuclear cells in myopathies. I: Quantitation of subsets according to diagnosis and sites of accumulation and demonstration and counts of muscle fibers invaded by T cells. Ann. Neurol..

[50] Brancaccio P., Lippi G., Maffulli N. (2010). Biochemical markers of muscular damage. Clin. Chem. Lab. Med..

[51] Kalluri R., LeBleu V.S. (2020). The Biology, Function, and Biomedical Applications of Exosomes. Science.

[52] Holl E.K., Roney K.E., Allen I.C., Steinbach E., Arthur J.C., Buntzman A., Plevy S., Frelinger J., Ting J.P. (2012). Plexin-B2 and Plexin-D1 in Dendritic Cells: Expression and IL-12/IL-23p40 Production. PLoS ONE.

[53] Carvalheiro T., Rafael-Vidal C., Malvar-Fernandez B., Lopes A.P., Pego-Reigosa J.M., Radstake T.R.D.J., Garcia S. (2020). Semaphorin4A-Plexin D1 Axis Induces Th2 and Th17 While Represses Th1 Skewing in an Autocrine Manner. Int. J. Mol. Sci..

[54] Chen Y., Shi R., Geraci N., Shrestha S., Gordish-Dressman H., Pachman L.M. (2008). Duration of chronic inflammation alters gene expression in muscle from untreated girls with juvenile dermatomyositis. BMC Immunol..

[55] Mazzotta C., Romano E., Bruni C., Manetti M., Lepri G., Bellando-Randone S., Blagojevic J., Ibba-Manneschi L., Matucci-Cerinic M., Guiducci S. (2015). Plexin-D1/Semaphorin 3E pathway may contribute to dysregulation of vascular tone control and defective angiogenesis in systemic sclerosis. Arthritis Res. Ther..

[56] Miranda K.C., Bond D.T., McKee M., Skog J., Păunescu T.G., Da Silva N., Brown D., Russo L.M. (2010). Nucleic acids within urinary exosomes/microvesicles are potential biomarkers for renal disease. Kidney Int..

[57] Cheng L., Sharples R.A., Scicluna B.J., Hill A.F. (2014). Exosomes provide a protective and enriched source of miRNA for biomarker profiling compared to intracellular and cell-free blood. J. Extracell. Vesicles.

[58] Wang J., Yue B., Huang Y., Lan X., Liu W., Chen H. (2022). Exosomal RNAs: Novel Potential Biomarkers for Diseases—A Review. Int. J. Mol. Sci..

[59] Misunova M., Salinas-Riester G., Luthin S., Pommerenke C., Husakova M., Zavada J., Klein M., Plestilova L., Svitalkova T., Cepek P. (2016). Microarray analysis of circulating micro RNAs in the serum of patients with polymyositis and dermatomyositis reveals a distinct disease expression profile and is associated with disease activity. Clin. Exp. Rheumatol..

[60] Peng Q., Zhang Y., Yang H., Shu X., Lu X., Wang G. (2016). Transcriptomic profiling of long non-coding RNAs in dermatomyositis by microarray analysis. Sci. Rep..

[61] Yakubovich E.I., Polischouk A.G., Evtushenko V.I. (2022). Principles and Problems of Exosome Isolation from Biological Fluids. Biochem. Mosc. Suppl. Ser. A.

[62] Zhang Q., Jeppesen D.K., Higginbotham J.N., Graves-Deal R., Trinh V.Q., Ramirez M.A., Sohn Y., Neininger A.C., Taneja N., McKinley E.T. (2021). Supermeres are functional extracellular nanoparticles replete with disease biomarkers and therapeutic targets. Nature.

[63] Zhang H., Freitas D., Kim H.S., Fabijanic K., Li Z., Chen H., Mark M.T. (2018). Identification of distinct nanoparticles and subsets of extracellular vesicles by asymmetric flow field-flow fractionation. Nat. Cell Biol..

[64] Jeppesen D.K., Zhang Q., Franklin J.L., Coffey R.J. (2023). Extracellular vesicles and nanoparticles: Emerging complexities. Trends Cell Biol..

[65] Arroyo J.D., Chevillet J.R., Kroh E.M., Ruf I.K., Pritchard C.C., Gibson D.F., Mitchell P.S., Bennett C.F., Pogosova-Agadjanyan E.L., Stirewalt D.L. (2011). Argonaute2 complexes carry a population of circulating microRNAs independent of vesicles in human plasma. Proc. Natl. Acad. Sci. USA.

[66] Li K., Wong D.K., Luk F.S., Kim R.Y., Raffai R.L. (2018). Isolation of Plasma Lipoproteins as a Source of Extracellular RNA. Extracellular RNA.

[67] Alinovskaya L.I., Sedykh S.E., Ivanisenko N.V., Soboleva S.E., Nevinsky G.A. (2018). How human serum albumin recognizes DNA and RNA. Biol. Chem..

[68] Vita G.M., De Simone G., De Marinis E., Nervi C., Ascenzi P., di Masi A. (2022). Serum albumin and nucleic acids biodistribution: From molecular aspects to biotechnological applications. IUBMB Life.

[69] Zhang Q., Jeppesen D.K., Higginbotham J.N., Franklin J.L., Coffey R.J. (2023). Comprehensive isolation of extracellular vesicles and nanoparticles. Nat. Protoc..

[70] Noguchi S., Tozawa S., Sakurai T., Ohkuchi A., Takahashi H., Fujiwara H., Takizawa T. (2024). BeWo exomeres are enriched for bioactive extracellular placenta-specific C19MC miRNAs. J. Reprod. Immunol..

[71] Cocozza F., Martin-Jaular L., Lippens L., Di Cicco A., Arribas Y.A., Ansart N., Dingli F., Richard M., Merle L., Jouve San Roman M. (2023). Extracellular vesicles and co-isolated endogenous retroviruses from murine cancer cells differentially affect dendritic cells. EMBO J..

[72] Torralba D., Baixauli F., Villarroya-Beltri C., Fernández-Delgado I., Latorre-Pellicer A., Acín-Pérez R., Martín-Cófreces N.B., Jaso-Tamame Á.L., Iborra S., Jorge I. (2018). Priming of dendritic cells by DNA-containing extracellular vesicles from activated T cells through antigen-driven contacts. Nat. Commun..

[73] Ruggeri Z.M. (2001). Structure of von Willebrand factor and its function in platelet adhesion and thrombus formation. Best Pract. Res. Clin. Haematol..

[74] Dent J.A., Galbusera M., Ruggeri Z.M. (1991). Heterogeneity of plasma von Willebrand factor multimers resulting from proteolysis of the constituent subunit. J. Clin. Investig..

[75] Mercuri E., Bönnemann C.G., Muntoni F. (2019). Muscular dystrophies. Lancet.

[76] Satoh M., Tanaka S., Ceribelli A., Calise S.J., Chan E.K.L. (2017). A Comprehensive Overview on Myositis-Specific Antibodies: New and Old Biomarkers in Idiopathic Inflammatory Myopathy. Clin. Rev. Allerg. Immunol..

[77] Betteridge Z., McHugh N. (2016). Myositis-specific autoantibodies: An important tool to support diagnosis of myositis. J. Intern. Med..

[78] Friedman A.W., Targoff I.N., Arnett F.C. (1996). Interstitial lung disease with autoantibodies againstaminoacyl-tRNA synthetases in the absence of clinically apparent myositis. Semin. Arthritis Rheum..

[79] Clawson K., Oddis C.V. (1995). Adult respiratory distress syndrome in polymyositis patients with the anti–jo-1 antibody. Arthritis Rheum..

[80] Sato S., Hoshino K., Satoh T., Fujita T., Kawakami Y., Fujita T., Kuwana M. (2009). RNA helicase encoded by melanoma differentiation–associated gene 5 is a major autoantigen in patients with clinically amyopathic dermatomyositis: Association with rapidly progressive interstitial lung disease. Arthritis Rheum. Off. J. Am. Coll. Rheumatol..

[81] Hoshino K., Muro Y., Sugiura K., Tomita Y., Nakashima R., Mimori T. (2010). Anti-MDA5 and anti-TIF1-γ antibodies have clinical significance for patients with dermatomyositis. Rheumatology.

[82] Chen F., Wang D., Shu X., Nakashima R., Wang G. (2012). Anti-MDA5 antibody is associated with A/SIP and decreased T cells in peripheral blood and predicts poor prognosis of ILD in Chinese patients with dermatomyositis. Rheumatol. Int..

[83] Sun Y.X., Wright H.T., Janciauskiene S. (2002). A β 1–42, α 1-antichymotrypsin, and their mixture. Cell. Mol. Life Sci. CMLS.

[84] Bodmer J.L., Schnebli H.P. (1984). Plasma proteinase inhibitors. Schweiz. Med. Wochenschr..

[85] Turnier J.L., Brunner H.I., Bennett M., Aleed A., Gulati G., Haffey W.D., Thornton S., Wagner M., Devarajan P., Witte D. (2019). Discovery of SERPINA3 as a candidate urinary biomarker of lupus nephritis activity. Rheumatology.

[86] Xu W., Liu X., Su L., Huang A. (2020). Association of MASP2 levels and MASP2 gene polymorphisms with systemic lupus erythematosus. J. Cell. Mol. Med..

[87] Asanuma Y., Nozawa K., Matsushita M., Kusaoi M., Abe Y., Yamaji K., Tamura N. (2023). Critical role of lectin pathway mediated by MBL-associated serine proteases in complement activation for the pathogenesis in systemic lupus erythematosus. Heliyon.

[88] Anyanwu C.O., Fiorentino D.F., Chung L., Dzuong C., Wang Y., Okawa J., Carr K., Propert K.J., Werth V.P. (2015). Validation of the Cutaneous Dermatomyositis Disease Area and Severity Index: Characterizing disease severity and assessing responsiveness to clinical change. Br. J. Dermatol..

[89] Liu M., Werth V.P., Williams K.J. (2020). Blood plasma versus serum: Which is right for sampling circulating membrane microvesicles in human subjects?. Ann. Rheum. Dis..

[90] Witwer K.W., Buzás E.I., Bemis L.T., Bora A., Lässer C., Lötvall J., Nolte-’t Hoen E.N., Piper M.G., Sivaraman S., Skog J. (2013). Standardization of sample collection, isolation and analysis methods in extracellular vesicle research. J. Extracell. Vesicles.

